# Transfersomes: A Promising Nanoencapsulation Technique for Transdermal Drug Delivery

**DOI:** 10.3390/pharmaceutics12090855

**Published:** 2020-09-09

**Authors:** Shakthi Apsara Thejani Opatha, Varin Titapiwatanakun, Romchat Chutoprapat

**Affiliations:** Department of Pharmaceutics and Industrial Pharmacy, Faculty of Pharmaceutical Sciences, Chulalongkorn University, Bangkok 10330, Thailand; 6272015933@student.chula.ac.th (S.A.T.O.); varin.t@pharm.chula.ac.th (V.T.)

**Keywords:** transfersomes, nanoencapsulation, transdermal drug delivery

## Abstract

Transdermal delivery systems have gained much interest in recent years owing to their advantages compared to conventional oral and parenteral delivery systems. They are noninvasive and self-administered delivery systems that can improve patient compliance and provide a controlled release of the therapeutic agents. The greatest challenge of transdermal delivery systems is the barrier function of the skin’s outermost layer. Molecules with molecular weights greater than 500 Da and ionized compounds generally do not pass through the skin. Therefore, only a limited number of drugs are capable of being administered by this route. Encapsulating the drugs in transfersomes are one of the potential approaches to overcome this problem. They have a bilayered structure that facilitates the encapsulation of lipophilic and hydrophilic, as well as amphiphilic, drug with higher permeation efficiencies compared to conventional liposomes. Transfersomes are elastic in nature, which can deform and squeeze themselves as an intact vesicle through narrow pores that are significantly smaller than its size. This review aims to describe the concept of transfersomes, the mechanism of action, different methods of preparation and characterization and factors affecting the properties of transfersomes, along with their recent applications in the transdermal administration of drugs.

## 1. Introduction

An efficacious, successful therapeutic treatment cannot be achieved in most cases, often due to many reasons, such as the occurrence of hepatic first-pass metabolism, adverse side effects, the rejection of invasive treatments and poor patient compliance [1]. Therefore, several drug delivery systems have been developed and studied over the past decades to overcome these problems. One promising approach is the use of transdermal delivery systems, as they are minimally invasive methods without first-pass effects. However, the barrier function of the skin that prevents or dampens the transdermal delivery of therapeutic agents has to be addressed [2,3]. Nanoencapsulation using a lipid-based vesicular system such as liposomes has been used to overcome the aforesaid challenge [4]. Liposomes facilitate drug transport through the skin by three possible mechanisms: adsorption to the skin surface with a subsequent transferring of the drug directly from vesicles to skin, fusion with the lipid matrix of the stratum corneum, thereby increasing drug partitioning into the skin, and lipid exchange between the liposomal membrane and cell membrane, facilitating the diffusion of the drug across the membrane [5,6]. However, the problem with conventional liposomes is that they do not deeply penetrate into the viable skin and blood circulation [7,8,9]. Therefore, liposomes have been widely used as drug carriers for dermal delivery and not for transdermal delivery. Conventional liposomes also have limitations, such as the poor encapsulation efficiency of hydrophilic drugs, an unstable membrane that results in leaky behavior and a short half-life [10,11,12]. These major obstacles have led to the discovery and development of other novel vesicles such as niosomes, sphingosomes, bilosomes, chitosomes, transfersomes, ethosomes and invasomes. Niosomes were first reported in the early 1970s. They are composed of nonionic surfactants (i.e., of alkyl or di-alkyl polyglycerol ether class, alkyl ethers, alkyl esters or alkyl amides); cholesterol and, sometimes, ionic amphiphiles. The cholesterol provides rigidity to the vesicular bilayer, whereas nonionic surfactants increase the size and entrapment efficiency of niosomes [13]. Furthermore, some ionic amphiphiles such as dicetyl phosphate (negatively charged molecule) and stearylamine (positively charged molecule) are used in the niosomes for enhancing the entrapment efficiency, efficacy and stability [14]. Moreover, they are identified as a better drug carrier system over liposomes due to considering factors such as a high chemical stability, high bioavailability, high entrapment efficiency and being inexpensive. According to the literature, niosomes tend to enhance the residence time of therapeutic drugs in the stratum corneum and epidermis, meanwhile reducing the systemic drug absorption and thereby improving the trapped drug penetration across the skin [15]. Chitosomes, liposomes-based vesicles coated with chitosan polymer, were first described in the early 1990s. Chitosan is used to improve the vesicular stability by modifying the surface properties of liposomes [16]. Moreover, chitosan provided mucoadhesive properties to the liposomes [17]. As mentioned by Mertins and Dimova in 2011, chitosan increases the membrane structural integrity of liposomes, decreases the membrane fluidity and thereby enhances the physicochemical stability of liposomes [18]. Sphingosomes were first introduced in the late 1990s. They can be identified as liposomes made from sphingolipids (such as lysoglycosphingolipids, hexadecasphinganine, n-acylsphingosines, phosphoglycosphingolipids and gangliosides) and cholesterol. Sphingosomes are more stable than phospholipid liposomes, because sphingolipids consist of only amide and ether linkages that are more hydrolysis-resistant than that of lecithin ester linkages. They also contain fewer double bonds than lecithin and, therefore, are less subjected to rancidity [19]. For bilosomes, they are vesicles composed of nonionic surfactants and bile salts (bile salts are incorporated into the niosome’s membrane), which are considered as non-lipoidal biocarriers. These novel vesicles were developed for the purpose of vaccine oral delivery due to its resistance towards the enzymes and bile salts in the gastrointestinal tract [20]. According to Yang et al. (2011), bile salts are considered as endogenous surfactants that are comprehensively used as absorption enhancers to improve the drug permeation across biological membranes [21]. A new type of carrier system—namely, transfersomes—were introduced by Cevc et al. in the 1990s. Transfersomes are composed of phospholipids and edge activator (EA), which is a membrane-softening agent (such as Tween 80, Span 80 and sodium cholate) that facilitates the ultra-deformable property of the transfersomes. When transfersomes reach the skin pores, they are capable of changing their membrane flexibility and passing through the skin pores spontaneously. This is the so-called self-optimizing deformability [22]. Moreover, transferomes are extremely deformable; therefore, they easily cross even the very narrow pores [23]. These self-optimizing, highly deformable lipid aggregates were successfully used in extensive preclinical tests and diverse arrays of phase I and phase II clinical trials, as well as for the transcutaneous delivery of peptides and proteins and the sustain release of desired therapeutic agents [8,24]. A number of transfersomes-based formulations are currently being assessed at different stages of clinical trials. For example, the study of the safety and efficacy of ketoprofen incorporated in transfersomes (Diractin^®^) for the treatment of osteoarthritis of the knees was carried out under a phase III clinical trial. It has been confirmed that the drug entrapped in transfersomal carriers exhibits superior therapeutic potency in relieving the pain of knee osteoarthritis over a six-week treatment period compared to a placebo and comparatively fewer adverse events [25]. The topical application of insulin-loaded transfersomes (Transfersulin^®^) for hypoglycemic effects are being investigated under a phase I clinical trial. In the preclinical study, Transfersulin^®^ could decrease the blood glucose level in alloxan-induced diabetic rabbits [26]. The risk–benefit ratio for topical triamcinolone acetonide in transfersomes in comparison with commercially available triamcinolone acetonide-containing cream and ointment were already assessed under a randomized controlled trial. It has been concluded that transfersomes may significantly improve the risk–benefit ratio of topical triamcinolone acetonide [27]. Therefore, transfersomes are known to be the most outstanding innovative transdermal drug carrier to this date [28]. These encouraging results seen with transfersomes led to the development of ethosomes and invasomes by playing around with vesicle compositions. Ethosomes are vesicles composed of phospholipids and relatively high amounts of ethanol (20–50%) and water. Due to the fact that ethosomes contains high ethanol concentrations, this causes alterations of the lipid bilayer of the skin and thereby enhances the stratum corneum penetration ability of the vesicles [29,30]. Invasomes are elastic phospholipid-based vesicles composed of phospholipids, terpenes and ethanol. Terpenes and ethanol have exhibited the ability to potentially improve the drug penetration across the stratum corneum tight lipid packing by the means of disrupting it [31,32]. The understanding of the specific term, underlying concept, preparation and characterization methods, as well as factors affecting the properties of the first generation of elastic vesicles (transfersomes), will be useful for the researchers getting into or conducting research in elastic vesicle-based transdermal drug delivery. In the current review, we provide an overview of the mechanisms of action, different preparations and characterization methods—factors affecting the properties of transfersomes, with particular emphasis on their recent applications in the transdermal administration of drugs.

## 2. Transfersomes

**Figure 1:**
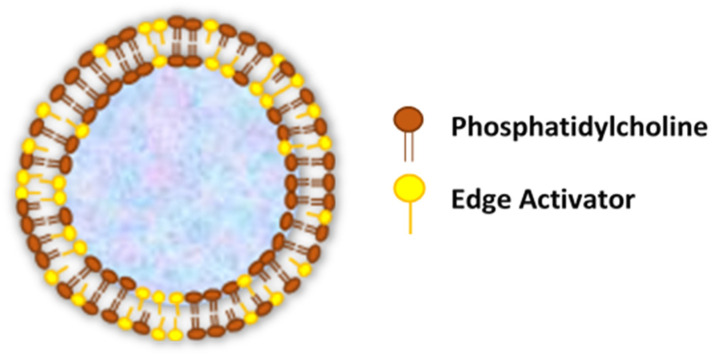
Structure of transfersomes.

Transferosomes are vesicular carrier systems that are specially designed to have at least one inner aqueous compartment that is enclosed by a lipid bilayer, together with an edge activator (Figure 1) [22].

This aqueous core surrounded by a lipid bilayer makes ultra-deformable vesicles having both self-optimizing and self-regulating capabilities [33]. In accordance with that, transferosomes are elastic in nature and can thereby deform and squeeze themselves as intact vesicles without a measurable loss through narrow pores or constrictions of the skin that are significantly smaller than the vesicle size [34,35]. In contrast to conventional liposomes, which are comprised of natural (such as egg phosphatidylcholine—EPC and soybean phosphatidylcholine—SPC) or synthetic (such as dimyristoyl phosphatidylcholine—DMPC, dipalmitoyl phosphatidylcholine—DPPC and dipalmitoyl phosphatidyl glycerol—DPPG) phospholipids [36], the modified liposomal vesicular system (transfersomes) is composed of the phospholipid component and single-chain surfactant as an edge activator [37]. Edge activators (EAs) function in an exceptional manner as membrane-destabilizing factors to increase the deformability of vesicle membranes and, when combined in a proper ratio with an appropriate lipid, gives the optimal mixture, enabling the transfersomes to become deformable, as well as ultra-flexible, which results in a higher permeation capability [38,39]. Therefore, transfersomes overcome the major drawbacks of conventional liposomes and penetrate pores that are much smaller than their own diameters. Furthermore, the transfersomes maintained their diameters against fragmentation, even after penetration through the smaller-sized pores. Due to the usage of EAs in the transfersomal formulation, it has achieved an enhanced performance compared to the conventional liposomes [38,39]. The EAs used in transfersomal formulations can also facilitate the solubilization of hydrophobic drugs, thereby increasing the drug entrapment efficiency of the formulations [40,41,42]. Moreover, the EAs have the potential to solubilize and fluidize the skin lipids, resulting in skin permeation enhancements [43,44]. The effect of EAs associated in skin permeations depends on their types and concentrations. Surfactants are one of many different compounds that act as edge activators and penetration enhancers [45]. They are known to be amphiphilic molecules that consist of a lipophilic alkyl chain that is connected to a hydrophilic head group [46]. Generally, rather than cationic surfactants, anionic surfactants are furthermore effective in enhancing the skin penetration, and the critical micelle concentration is also lower, whereas nonionic surfactants with an uncharged polar head group are better-tolerated than cationic and anionic surfactants [45]. Nonionic surfactants are considered less toxic and less hemolytic, as well as less irritating to cellular surfaces, and they tend to maintain a near physiological pH in a solution. Moreover, they have various functions, including acting as solubilizers, emulsifiers and strong P-glycoprotein inhibitors, which is useful for enhancing the drug absorption, as well as for targeting specific tissues [46]. Transfersomal formulations are highly utilized in “peripheral drug targeting”, “transdermal immunization” and well-recognized to be a major system for the “transdermal delivery” of a vast array of therapeutic agents [47]. According to various research publications, it is evidently known that transfersomes are capable of transporting low, as well as high, molecular weight (200 ≤ MW ≤ 10^6^) bioactive molecules and hydrophilic and lipophilic molecules through the skin with a transport efficiency greater than 50%. The certain arrangement of cells in the skin prevents appreciable molecule exchanges between the skin surface and the skin depth. Even water can only penetrate through the skin barrier at the rate of only 0.4 mg/cm^2^/h. It was found that the transcutaneous permeation rates measured for the molecules with molecular weights between 18 Da (water) and 750 Da (drugs) were between 0.1 g/h/cm^2^ and 1 µg/h/cm^2^ at most, and the transferomes were capable of transporting these drug molecules through the skin of more than 50%, in comparison with the unentrapped drug [7]. Moreover, it has been reported that transfersomes can significantly transfer lipophilic fluorescence markers through the murine skin of more than 50% compared to that of liposomes [7]. The presence of lipophilic and hydrophilic moieties in the vesicular structure result in wide range of solubility of transfersomes [7,48,49]. It has been identified that vesicles with sizes ≥600 nm are unable to penetrate deeper skin layers, whereas ≤300 nm reach deeper into the skin [50,51]. However, those vesicles with ≤70 nm have exhibited a maximum deposition of the contents in both the viable epidermal as well as the dermal layers of the skin. Furthermore, it has been reported that statistically enhanced skin penetration was exhibited by transfersomes with 120-nm sizes compared to larger ones [52]. The optimized delivery, improved bioavailability and promising stability of phytoactives in herbal formulations are also ensured by these novel transfersome delivery systems [53]. Therefore, herbal ingredients can be encapsulated in transfersomes to supply promising skin care and various therapeutic benefits [47]. It was described in several published research papers that, due to vast skin-penetration capabilities, transfersomes are able to create skin drug depots for a sustained drug release, deliver therapeutic agents into deeper skin layers or transport drugs into the systemic circulation [54]. Hence, transfersomes propose a good potential to provide a whole new perspective for the rational drug delivery [55].

Advantages of Transfersomes as Vesicle-based Transdermal Drug Delivery Systems [2,36,56,57]:

- Transfersomes carriers are composed of hydrophilic and hydrophobic moieties, which result in becoming a unique drug carrier system that can deliver therapeutic agents with wide range of solubility.
- Transfersomes are able to squeeze themselves through constrictions of the skin barrier that are very narrow, such as 5 to 10 times less than the vesicle diameter, owing to their ultra-deformability and elastic properties.
- High vesicle deformability facilitates the transport of drugs across the skin without any measurable loss in intact vesicles and can be used for both topical, as well as systemic, treatments.
- Transfersomes carriers are very versatile and efficient in accommodating a variety of agents nearly independent of their size, structure, molecular weight or polarity.
- They are made up of natural phospholipids and EAs, therefore promisingly biocompatible and biodegradable.
- Transfersomes can be used for the delivery of various active compounds, including proteins and peptides, insulin, corticosteroids, interferons, anesthetics, NSAIDs, anticancer drugs and herbal drugs.
- Transfersomes are an obvious choice for achieving a sustained drug release, as well as a predictable and extended duration of activity.
- They are capable of increasing the transdermal flux and improving the site specificity of bioactive agents.
- Avoiding the first-pass metabolism, which is a major drawback in oral drug administration, and result in optimized bioavailability of the drug.
- Minimize the undesirable side effects of the drug, as well as protect the drug from metabolic degradation; moreover, the utility of short half-life drugs.
- In most of the cases, a relatively high entrapment efficiency (EE) of nearly 90% of the lipophilic drug can be achieved by transfersomes. For transfersome formulations of diclofenac diethylamine (DDEA) and curcumin (CRM), the maximum entrapment efficiency achieved was over 90% for both DDEA and CRM transfersomes. Nevertheless, the entrapment efficiency can variate due to various reasons, as, when the lipid concentration was more, a high entrapment efficiency could be observed. The EE decreases when the surfactant concentration increases above certain concentrations due to the formation of mixed micelles. According to the literature, in case of a low EE, the lipophilic drug encapsulation could be enhanced by incorporating a surfactant with a low HLB (hydrophilic-lipophilic balance) value. It has been identified as a fact that transfersomes show a distinctive property of the very high encapsulation of lipophilic drugs.
- They have the advantage of being made from pharmaceutically acceptable ingredients using standard methods but need to be designed and optimized on a case-by-case basis.
- Due to a short and simple production procedure, it is easy to scale-up.

Limitations of transfersomes [2,36,58]:

- Transfersomes are considered as chemically unstable due to their tendency to oxidative degradation. The oxidation of transfersomes can be significantly decreased when the aqueous media is degassed and purged with inert gases, such as nitrogen and argon [59]. Storage at a low temperature and protection from light will also reduce the chance of oxidation [60]. Post-preparation processing, such as freeze-drying and spray-drying, can improve the storage stability of transfersomes [61].
- Another obstacle of utilizing transfersomes as a drug delivery system is the difficulty to achieve the purity of natural phospholipids. Therefore, synthetic phospholipids could be used as alternatives [62].
- The expensiveness of transfersomal formulations is associated with the raw materials used in lipid excipients, as well as the expensive equipment needed to increase manufacturing. Hence, the widely used lipid component is phosphatidylcholine, because it is relatively low in cost [6].

## 3. Mechanism of Action

**Figure 2:**
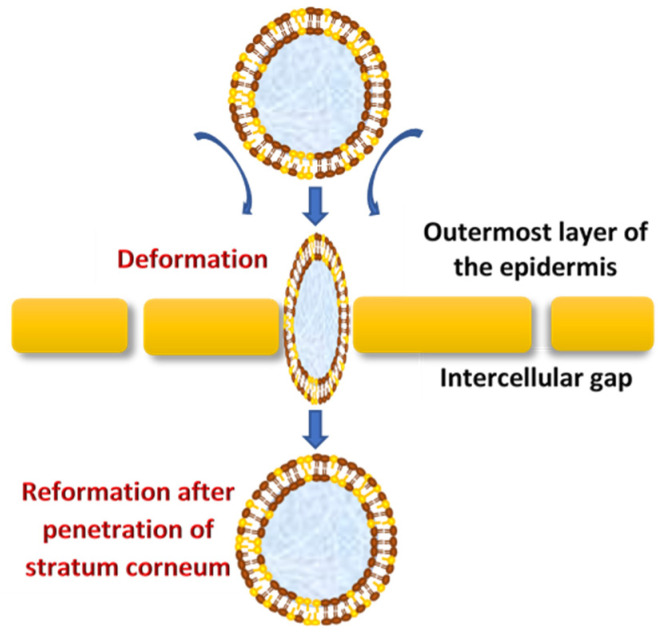
The mechanism of action of transfersomes.

Vesicles are known as colloidal particles, which are an aqueous compartment enclosed by a concentric bilayer that are made-up of amphiphilic molecules. They are very useful as vesicular drug delivery systems, which transport hydrophilic drugs encapsulated in the inner aqueous compartment, whereas hydrophobic drugs are entrapped within the lipid bilayer [47]. With regard to transfersomes, they are highly deformable (ultra-flexible) and self-optimizing novel drug carrier vesicles, in which their passage across the skin is mainly associated with the transfersomes’ membrane flexibility, hydrophilicity and the ability to maintain the vesicle’s integrity (Figure 2) [63,64].

They efficiently penetrate through the intact skin if applied under nonocclusive conditions; this specific nonocclusive state of the skin is required mainly to initiate a transepidermal osmotic gradient across the skin [1,54]. According to the study done by Cevc and Blume, hydrotaxis (xerophobia) is the permeation mechanism of transfersomes, which is further described as the transferosome’s moisture-seeking tendency towards deeper skin layers rather than dry outer background due to the state of moisture evaporation from the transfersomal formulation following its application on the skin (nonocclusive condition) [65]. The transdermal water activity difference, which originates due to the natural transdermal gradient, creates a significantly strong force that acts upon the skin through transfersomes vesicles, which enforce the widening of intercellular junctions with the lowest resistance and thereby generate transcutaneous channels 20–30 nm in width. These created channels allow the transfer of ultra-deformable, slimed transfersomes across the skin with respect to the hydration gradient [23]. Moreover, the osmotic gradient develops as a result of evaporation of the skin surface water due to body heat, which exerts its action as the driving force to facilitate the flexible transport across the skin to deliver therapeutic agents from the site of application to the target area for local or systemic treatments in effective therapeutic concentrations and minimum systemic toxicity [33]. Transfersomes demonstrate a higher permeation efficiency (through small skin channels) compared to conventional liposomes but have a similar bilayered structure that facilitates the encapsulation of lipophilic and hydrophilic, as well as amphiphilic, drugs [53]. Transfersomes vary from liposomes, primarily due to their softer, better adjustable and ultra-deformable artificial membranes. Interdependency of the local composition, as well as the shape of the lipid bilayer, makes the vesicles both self-optimizing and self-regulating. This property enables the transfersomes vesicles to cross numerous transport barriers efficiently. Therefore, transfersomes are supramolecular entities composed of at least one type of amphipathic agent and, by the addition of at least one type of bilayer-softening agent (edge activator), result in greatly increased lipid bilayer flexibility and permeability [8]. Certain transfersomes have some amounts of alcohol (ethanol or propylene glycol) in their compositions as penetration enhancers and, also, used as cosolvents that have good solvating power. Ethanol has been proposed to induce modifications of the lipid bilayer polar head region. Following penetration, ethanol increases the fluidity of the intercellular lipid matrix and later on results in decreasing the density of the lipid lamellae [46]. Transfersomes can penetrate through the stratum corneum and reach the target sites, including the dermis and blood circulation. Their penetration ability depends on the deformability of the transfersomal membrane, which can be attributed to the vesicle compositions [66,67]. Therefore, the most suitable vesicle compositions must be identified through conducting individually designed experimental procedures for each therapeutic agent to obtain the most appropriate carriers with optimum deformability, drug carrying capacity and stability.

## 4. Composition of Transfersomes

Transfersomes are generally composed of

- firstly, the main ingredient, an amphipathic ingredient (e.g., soy phosphatidylcholine, egg phosphatidylcholine, etc.) that can be a mixture of lipids, which are the vesicle-forming components that create the lipid bilayer [52,61].
- secondly, 10–25% surfactants/edge activators; the most commonly used edge activators in transfersome preparations are surfactants as sodium cholates; sodium deoxycholate; Tweens and Spans (Tween 20, Tween 60, Tween 80, Span 60, Span 65 and Span 80) and dipotassium glycyrrhizinate, which are biocompatible bilayer-softening compounds that increase the vesicles’ bilayer flexibility and improve the permeability [3,8,52,68,69].
- about 3–10% alcohol (ethanol or methanol), as the solvent and, finally, hydrating medium consist with either water or a saline phosphate buffer (pH 6.5–7) [48,55].

In an aqueous environment, the phospholipids self-assemble into flexible lipid bilayers and close to form vesicles [7]. The biocompatible membrane softeners, which are also known as edge activators, are single-chain surfactants that incorporate into the transfersomes structure and facilitate the destabilization of the vesicle’s lipid bilayer and enhance its fluidity and elasticity [4,37,65,70]. The total amount of surfactants and the proper ratios of individual surfactants to phospholipids are responsible for the control of vesicles’ membrane flexibility and minimizing the risk towards vesicle ruptures in the skin [71]. This result promotes transfersomes to follow the natural osmotic gradient across the epidermis following application under a nonocclusive manner [33,72]. In summary, the penetration-enhancing effect of these vesicles depends on the concentrations and the types of surfactants, the types of lipids, the size shape and elasticity of the vesicles.

## 5. Preparation Methods

Even though there are various patented procedures of transfersome preparation, there is no general preparation protocol or a specific formula for this process [73,74]. Therefore, the best preparation conditions and vesicles compositions must be identified, designed and optimized through conducting individually designed experimental procedures for each therapeutic agent to obtain the most appropriate carriers with optimum deformability, drug carrying capacity and stability [28]. The conventional method of transfersome preparation is the thin film hydration technique, also known as the rotary evaporation-sonication method. Other modified preparation methods are vortexing-sonication, the modified handshaking process, suspension homogenization, centrifugation process, reverse-phase evaporation method, high-pressure homogenization technique and ethanol injection method. Each method is generally described as follows:

### 5.1. Thin Film Hydration Technique/Rotary Evaporation-Sonication Method

The phospholipids and edge activator (vesicle-forming ingredients) are dissolved in a round-bottom flask using a volatile organic solvent mixture (example: chloroform and methanol in a suitable (*v/v*) ratio). The lipophilic drug can be incorporated in this step. In order to form a thin film, the organic solvent is evaporated above the lipid transition temperature under reduced pressure using a rotary vacuum evaporator. Keep it under vacuum to remove the final traces of the solvent. The deposited thin film is then hydrated using a buffer solution with the appropriate pH (example: pH 7.4) by rotation for a respective time at the corresponding temperature. The hydrophilic drug incorporation can be done in this stage. The resulting vesicles are swollen at room temperature and sonicated in a bath or probe sonicator to obtain small vesicles. The sonicated vesicles are homogenized by extrusion through a sandwich of 200 nm to 100 nm polycarbonate membranes [2,75].

### 5.2. Vortexing-Sonication Method

The phospholipids, edge activator and the drug are mixed in a phosphate buffer. The mixture is then vortexed until a milky transfersomal suspension is obtained. It is then sonicated, using a bath sonicator, for a respective time at room temperature and then extruded through polycarbonate membranes (example: 450 and 220 nm) [76,77].

### 5.3. Modified Handshaking Process

The modified handshaking method has the same basic principle as the rotary evaporation-sonication method. In the modified handshaking process, the organic solvent, the lipophilic drug, the phospholipids and edge activator are added in a round-bottom flask. All the excipients should completely dissolve in the solvent and obtain a clear transparent solution. Then, the organic solvent is removed by evaporation while handshaking instead of using the rotary vacuum evaporator. In the meantime, the round-bottom flask is partially immersed in the water bath maintained at a high temperature (example: 40–60 °C). A thin lipid film is then formed inside the flask wall. The flask is kept overnight for complete evaporation of the solvent. The formed film is then hydrated with the appropriate buffer solution with gentle shaking at a temperature above its phase transition temperature. The hydrophilic drug incorporation can be done in this stage [75].

### 5.4. Suspension Homogenization Method

Transfersomes are prepared by mixing an ethanolic phospholipid solution with an appropriate amount of edge activator. The prepared suspension is subsequently mixed with buffer to yield a total lipid concentration. The resulting formulation is then sonicated, frozen and thawed respectively two to three times [1,49].

### 5.5. Centrifugation Process

The phospholipids, edge activator and the lipophilic drug are dissolved in the organic solvent. The solvent is then removed using a rotary evaporator under reduced pressure at the respective temperature. The remaining traces of solvent are removed under vacuum. The deposited lipid film is hydrated with the appropriate buffer solution by centrifuging at room temperature. The hydrophilic drug incorporation can be done in this stage. The resulting vesicles are swollen at room temperature. The obtained multilamellar lipid vesicles are further sonicated at room temperature [1].

### 5.6. Reverse-Phase Evaporation Method

The phospholipids and edge activator are added to a round-bottom flask and dissolved in the organic solvent mixture (example: diethyl ether and chloroform). The lipophilic drug can be incorporated in this step. Then, the solvent is evaporated using rotary evaporator to obtain the lipid films. The lipid films are redissolved in the organic phase mostly composed of isopropyl ether and/or diethyl ether. Subsequently, the aqueous phase is added to the organic phase, leading to a two-phase system. The hydrophilic drug incorporation can be done in this stage. This system is then subjected to sonication using a bath sonicator until a homogeneous w/o (water in oil) emulsion is formed. The organic solvent is slowly evaporated using rotary evaporator to form a viscous gel, which then becomes a vesicular suspension [11,78].

### 5.7. High-Pressure Homogenization Technique

The phospholipids, edge activator and the drug are uniformly dispersed in PBS or distilled water containing alcohol and followed by ultrasonic shaking and stirred simultaneously. The mixture is then subjected to intermittent ultrasonic shaking. The resulting mixture is then homogenized using a high-pressure homogenizer. Finally, the transfersomes are stored in appropriate conditions [7,79].

### 5.8. Ethanol Injection Method

The organic phase is produced by dissolving the phospholipid, edge activator and the lipophilic drug in ethanol with magnetic stirring for the respective time, until a clear solution is obtained. The aqueous phase is produced by dissolving the water-soluble substances in the phosphate buffer. The hydrophilic drug incorporation can be done in this stage. Both solutions are heated up to 45–50 °C. Afterwards, the ethanolic phospholipid solution is injected dropwise into the aqueous solution with continuous stirring for the respective time. Ethanol removal is done by transferring the resultant dispersion into a vacuum evaporator and then sonicating for particle size reduction [80,81].

## 6. Factors Affecting Properties of Transfersomes

In the process of obtaining an optimized formulation of transfersomes, there are number of process variables that could affect the properties of the transfersomes. These variables basically involve the manufacturing of transfersomal formulations, which are identified as follows:

### 6.1. Effect of Phospholipids: Edge Activator Ratio

The phospholipid: Edge activator (lecithin:surfactant) should be an optimized ratio due to the fact that this greatly affects the entrapment efficiency, vesicle size and permeation ability. In general, it has been reported that the EE could be reduced due to the presence of a higher surfactant concentration. This may be due to the result of increased vesicles’ membrane permeability because of the arrangement of surfactant molecules within the vesicular lipid bilayer structure, which could generate pores within the vesicular membrane and lead to an increased fluidity and prompt the leakage of the entrapped drug [57]. A further increase in the edge activator content may lead to pore formation in the bilayer and a reduced permeation ability of the vesicles [54], whereas the incorporation of low concentrations of surfactants may result in growth of the vesicle size. In addition, the decrease in vesicles size at high phospholipid concentrations has been reported in various studies [82,83].

### 6.2. Effect of Various Solvents

Various solvents such as ethanol or methanol are used. Selection of the appropriate solvent depends on the solubility of all the formulation ingredients in the solvent and their compatibility with the solvent. Preferably, all the excipients, including the drug, should completely dissolve in the solvent and should obtain a clear transparent solution to produce a better film-forming ability and good stability after hydration [76]. Solvents used in the formulation can also exert their function as penetration enhancers that improve drug flux through the membrane. According to Williams and Barry (2004), ethanol was used in various studies to enhance the flux of hydrocortisone, 5-fluorouracil, estradiol and levonorgestrel through rat skin [84]. For an example, ethanol increases the permeation through different mechanisms, such as increasing the drug solubility in vesicles by acting as a solvent, moreover permeating into the stratum corneum and altering the solubility properties of the respective tissue and, consequently, improving the drug partitioning into the membrane. Increasing the ethanol concentration in the formulation may result in a decrease in the %EE, which could be attributed to the increased permeability of the vesicular phospholipid bilayer. This may promote the consequent leakage of the encapsulated drug [85].

### 6.3. Effect of Various Edge Activators (Surfactants)

Deformability, as well as the entrapment efficiency of transfersome vesicles, are affected by the type of edge activators used in their formulations. This could be due to the difference in the chemical structure of the EA [53]. Generally, the vesicle size decreases by increasing the surfactant concentration, the hydrophilicity of the surfactant head group, carbon chain length and the hydrophilic lipophilic balance (HLB). The three surfactants, including tween 80, span 80 and sodium deoxycholate, were used to prepare the transfersomes, and a reduction of the vesicle size was found when the higher surfactant concentration used. This might be due to the fact that the high surfactant concentrations (more than 15%) induce micelle formation rather than vesicle formation [86]. A small polydispersity index (PDI) was reported with the higher surfactant concentration. A small PDI is responsible for consistent size distribution, which is thought to be an important factor for the reduction of interfacial tension and provides a homogeneous formulation. Additionally, an increased surfactant concentration may lead to an increase in charge of the vesicles, which results in a reduction of vesicle aggregation and enhances the stability of the system. In addition, surfactant properties are one of the properties that are responsible for the entrapment efficiency of the vesicles, as, for an example, the entrapment of a lipophilic drug would be enhanced with the use of a surfactant with a low HLB value. Moreover, it has been reported that higher surfactant concentrations will increase the formation of the vesicle number, which leads to a higher volume of the hydrophobic bilayer domain that is available for the entrapment of hydrophobic drugs. However, if the amount of lipophilic drug exceeds the vesicular loading capacity, it may disrupt the vesicular membrane, leading to drug leakage, lowering the entrapment efficiency and skin permeation ability [81]. Furthermore, the membrane permeability of vesicles depends on the carbon chain length and transition temperature of the surfactant. The optimum amount of surfactant used in the formulation depends on the packing density of the phospholipid used and the surfactant-phospholipid interaction [57]. The presence of surfactants can have an impact on the permeation property of transfersomes. According to a study by Cipolla et al. (2014), the amount of drug (ciprofloxacin) released was dependent on the concentration, as well as the type of surfactant used, and using Tween 80 significantly increased the release [66].

### 6.4. Effect of the Hydration Medium

The hydrating medium may consist of either water or saline phosphate buffer (pH 6.5–7). The pH level of the formulation should be suitable to achieve a balance between both the formulation properties and biological applications, as well as the route of administration. The lipid bilayer of transfersomes mimics the phospholipid layer of the cell membrane, and only unionized drugs remain membrane-bound to the phospholipid bilayer and penetrate through the intracellular route [67,87]. It is important to use the suitable pH of the hydration medium, which keeps the drug unionized to increase the entrapment and permeation of the drug.

## 7. Characterization of the Transfersomes

There are several published methods used to determine the characterization parameters of the transfersomes, such as the vesicle shape and size, size distribution, polydispersity index, zeta potential, number of vesicles for cubic mm, entrapment efficiency, degree of deformability and skin permeability measurements [2,50,69], which are beneficial for the optimization of the transfersomal formulation. Each characterization method mentioned above is explained in detail below.

### 7.1. Vesicle Size, Zeta Potential and Morphology

The vesicle size is one of the important parameters during transfersome preparation, batch-to-batch comparison and scale-up processes. During storage, the changing of the vesicle size is an important variable in terms of the physical stability of the formulation. Vesicles smaller than 40 nm are prone to fusion processes because of the high curvature state of their bilayer membranes, whereas much larger and electroneutral transfersomes are aggregated through van der Waals interactions due to relatively greater membrane contact areas. Vesicle size is a factor that influences the ability to encapsulate the drug compounds in transfersomes. For lipophilic and amphiphilic agents, a high lipid-to-core ratio is favored, while a larger aqueous core volume is preferred for the encapsulation of hydrophilic compounds. Generally, the dynamic light scattering (DLS) method or photon correlation spectroscopy (PCS) can be used to determine the vesicle diameter. The vesicle’s suspension can be mixed with an appropriate medium, and the vesicular size measurements can be obtained in triplicate. Moreover, as another approach, the sample can be prepared in distilled water and filtered through a 0.2 mm membrane filter. The filtered sample is then diluted with filtered saline to measure the size of the vesicles by DLS or PCS. Moreover, the DLS method-associated computerized inspection system by Malvern Zetasizer can be used for the determination of the vesicle size and size distribution, whereas the structural changes are observed by transmission electron microscopy (TEM). The zeta potential is measured by the electrophoretic mobility technique using Malvern Zetasizer. The visualization of transfersome vesicles can be done by using the phase contrast microscopy or TEM [1,7,53].

### 7.2. Number of Vesicles Per Cubic mm

This parameter is important for the optimization of the composition of the transfersomes and other process variables [1,3,53]. Unsonicated transfersomal formulations are diluted five times using 0.9% sodium chloride. A hemocytometer with an optical microscope is used to study this sample. The transfersomes with a vesicle size of more than 100 nm can be observed by optical microscope [88,89]. The number of transfersomes in small squares are counted and calculated using the following formula:

(1) $$\begin{array}{l} \mathrm{Total}\;\mathrm{number}\;\mathrm{of}\;\mathrm{transfersomes}\;\mathrm{per}\;\mathrm{cubic}\;\mathrm{mm}\\ \;=\;\frac{\left(\mathrm{Total}\;\mathrm{number}\;\mathrm{of}\;\mathrm{transfersomes}\;\mathrm{counted}\;\times \;\mathrm{dilution}\;\mathrm{factor}\;\times 4000\right)}{\mathrm{Total}\;\mathrm{number}\;\mathrm{of}\;\mathrm{squares}\;\mathrm{counted}} \end{array}$$

### 7.3. Entrapment Efficiency (%EE)

The entrapment efficiency (%EE) is the amount of drug entrapped in the formulation. The EE is determined by separating the unentrapped drug from the vesicles using various techniques, such as mini-column centrifugation. In this process, direct or indirect methods can be used to determine the %EE. After ultracentrifugation, the direct approach would be removing the supernatant followed by disrupting the sedimented vesicles using a suitable solvent that is capable of lysing the sediment. Subsequently, the resulting solution can be diluted and filtered using a syringe filter (0.22–0.45 µm) to remove the impurities. The drug content is determined by employing analytical methods, such as modified high-performance liquid chromatography (HPLC) or spectrophotometrically, which depends on the analytical method of the active pharmaceutical ingredient (API) [83,90,91]. The percentage drug entrapment (the entrapment efficiency) is expressed as:

(2) $$\%\mathrm{Entrapment}\;\mathrm{efficiency}\;=\;\frac{\mathrm{Amount}\;\mathrm{of}\;\mathrm{the}\;\mathrm{drug}\;\mathrm{entrapped}}{\mathrm{Total}\;\mathrm{amount}\;\mathrm{of}\;\mathrm{the}\;\mathrm{drug}\;\mathrm{added}}\times 100$$

The indirect approach to determine the %EE is diluting the supernatant using a suitable solvent and filtering it to remove the impurities. The concentration of the drug in the supernatant is determined as the free drug by an appropriate analytical method. Thereby, the percentage drug entrapment is expressed as:

(3) $$\%\mathrm{Entrapment}\;\mathrm{efficiency}\;=\;\frac{\mathrm{Total}\;\mathrm{amount}\;\mathrm{of}\;\mathrm{the}\;\mathrm{drug}\;\mathrm{added}\;-\;\mathrm{Amount}\;\mathrm{of}\;\mathrm{the}\;\mathrm{free}\;\mathrm{drug}}{\mathrm{Total}\;\mathrm{amount}\;\mathrm{of}\;\mathrm{the}\;\mathrm{drug}\;\mathrm{added}}\times 100$$

### 7.4. Degree of Deformability

This parameter is important, as it affects the permeation of the transfersomal formulation. This study is done using pure water as the standard. The preparation is passed through many microporous filters of known pore sizes between 50 to 400 nm. The particle size, as well as the size distribution, are noted after each pass using DLS measurements [1,3,53]. The degree of deformability is expressed as:

(4) $$D=J\left(\frac{\mathrm{rv}}{\mathrm{rp}}\right)$$

where D = degree of deformability, J = amount of suspension extruded during 5 min, rv = size of the vesicle and rp = pore size of the barrier.

### 7.5. In Vitro Drug Release

The in vitro drug release profile can provide fundamental information on the formulation design and details on the release mechanism and kinetics, enabling a scientific approach to optimize the transfersomal formulation. The in vitro drug release of transfersomes is typically evaluated in comparison to the free drug or the reference product. Various research studies have evidently provided successful data related to the drug release profiles of developed transfersome formulations. Celecoxib transfersomal gel for the rheumatoid arthritis treatment showed the release of 75% of the entrapped drug within 6 h, which is more than a 30% increment relative to the commercial gel [92]. Ketoconazole-loaded transfersomal gel showed an initial burst of the drug release of 40.67%, which was higher than that of ketoconazole suspension (27.35%) after 6 h [93]. The in vitro release profile of lidocaine from the transfersomal vesicles showed more than 80% of the drug released after 6 h [94]. In brief, Franz diffusion cells are employed in the in vitro drug release study. The donor chamber is fixed to the receptor chamber by means of adhesive tape. The fluid in the receptor chamber is constantly stirred by a magnetic bar. As normal skin surface temperature is approximately 32 °C [95], therefore, in the release study, the temperature of the receptor fluid should be kept at the in vivo skin surface temperature of 32 ± 1 °C [38,96]. A mixed cellulose ester membrane of an average pore size of 0.45 µm is used. The membranes are soaked in the release media (phosphate buffer) at room temperature overnight in order to allow the membrane pores to swell. The aliquots of 1 mL of the receptor medium are withdrawn at appropriate time intervals (such as 0, 0.5, 1, 2, 3, 4, 5 and 6 h), and simultaneously, the receptor medium is replaced by an equal volume of the fresh PBS to maintain the sink conditions. The obtained samples can be analyzed by using appropriate methods such as UV, HPLC and high-performance thin layer chromatography (HPTLC) [1,3,96].

### 7.6. In Vitro Skin Permeation Studies

This study is performed to determine the transport efficiencies of the transdermal delivery systems and identify the factors that increase the transdermal flux of the drugs, which is typically expressed in units of μg/cm^2^/h [97]. The information obtained from this study can also be used to predict in vivo behaviors from different transdermal delivery systems and used for the optimization of the formulation prior to performing more expensive in-vivo studies. Ideally, the human skin should be used for the evaluation of permeation properties of candidate formulations. However, the limited availability, ethical problems and religious restrictions of the human skin make it less attractive for the permeation study. Various animal models, such as primate, porcine, rat, mouse, guinea pig and snake skins, have been suggested as more accessible substitutes for human skin. However, it should be noted that percutaneous absorption through various animal skins may differ significantly from the results obtained with human skin models. According to the published data, it is evidently suggested that pig skin is the most suitable animal model for human skin due to the fact that the fluxes through the skin, as well as the concentrations in the skin, were exhibited to be of the same order of magnitude for both of those tissues, with minor differences of, at most, two or four-fold, respectively [98]. Moreover, as another option, synthetic membranes (example: Strat M^®^) have been employed in transdermal permeation studies. It has been reported that synthetic membranes show very close correlations to human skin. This model has the advantage as being more consistent in permeability, as well as responsiveness, in comparison with human and animal skins [99]. In brief, Franz diffusion cells are employed in the skin permeation study. The selected membranes are horizontally mounted on the receptor compartments as the side, indicating the stratum corneum facing upwards toward the donor compartments. The receptor compartments of the Franz diffusion cells are filled with phosphate buffer saline solution, which is stirred by a magnetic bar. As the receptor fluid is used to mimic blood circulation beneath the skin, the temperature of the receptor fluid should be kept at 37 ± 0.5 °C [40,93]. An appropriate amount of the testing formulation is added into each donor compartment as it is placed on the membrane, and the top of the diffusion cell is opened to mimic nonoccluded conditions. Specific volumes of aliquots of the receptor medium are withdrawn at appropriate time intervals, and simultaneously, the receptor medium is replaced by an equal volume of the fresh receptor medium to maintain the sink conditions. The obtained samples can be analyzed by HPLC or the spectroscopic method [1,48].

### 7.7. Stability of Transfersomes

The stability of transfersome vesicles can be determined by assessing the structure and the size of vesicles with respect to time. DLS and TEM can be used for the determination of the mean size and structural changes, respectively. The optimized transfersomal formulations can be stored in tightly sealed amber vials at different temperature conditions. According to ICH (International Conference on Harmonization) guidelines, under the stability testing of new drug substances and products, the general case for the storage condition is described as, for the long term, 25 ± 2 °C/60% relative humidity (RH) ± 5% RH or 30 ± 2 °C/65% RH ± 5% for 12 months and, for accelerated testing, 40 ± 2 °C/75% RH ± 5% for six months. Drug products intended for refrigeration should be subjected to long-term storage at a condition of 5 ± 3 °C for 12 months and accelerated study for 25 ± 2 °C/60% RH ± 5% RH for six months. A significant change for the drug product is defined as the failure to meet its specifications.

The parameters and the testing methods are summarized in the following (Table 1):

## 8. Applications of Transfersomes as the Transdermal Delivery System

Over the past decades, the applications of the transfersomes in the field of transdermal drug administration have been extensively studied. Some of these applications are described in the section below.

### 8.1. Delivery of Antioxidants

In 2017, Avadhani et al. developed nanotransfersomes containing epigallocatechin-3-gallate (EGCG) and hyaluronic acid by using a modified thin-film hydration method followed by the high-pressure homogenization technique in order to enhance their efficacies as UV radiation protectors, antioxidants and antiaging substances [100]. In 2019, Wu et al. prepared transfersomes combined with resveratrol using the high-pressure homogenization technique. It was found that the obtained transfersomes could improve the stability, bioavailability, solubility and safety of resveratrol [79].

### 8.2. Delivery of Anticancer Drugs

A research conducted by Jiang et al. in 2018 was associated with the topical chemotherapy of melanoma by transfersome-embedded oligopeptide hydrogels containing paclitaxel prepared by the thin-film dispersion method. Transfersomes composed of phosphatidylcholine, tween80 and sodium deoxycholate were shown to effectively penetrate into tumor tissues [52].

### 8.3. Delivery of Corticosteroids

The biological activity and characteristics of halogenated corticosteroid triamcinolone-acetonide-loaded transfersomes prepared by the conventional thin-film hydration technique were studied by Cevc and Blume in 2003 and 2004. The results showed that transfersomes had increased the biological potency and prolonged effect, as well as the reduced therapeutic dosage [101,102].

### 8.4. Delivery of Anti-Inflammatory Drugs

Diclofenac sodium, celecoxib, mefenamic acid and curcumin-loaded transfersomes were developed and studied for the purpose of topical administration by several research groups. Research findings suggested that transferomes could improve the stability and efficacy of the anti-inflammatory drugs [76,103,104].

In addition to the above information, brief details of various preparation and formulation aspects and the potential of transfersomes are mentioned in Table 2.

## 9. Conclusions

Transfersomes are ultra-deformable carriers that facilitate the delivery of a diverse array of drug molecules across the skin barrier with superior efficacy compared to the conventional vesicular systems. The osmotic gradient is the main driving force for the transport of transfersomes into the deeper skin layers. Importantly, transferosomes are specifically designed vesicular systems that need to be optimized in accordance with individual cases of drugs of interest to achieve the most effective formulations and ultimate pharmacological responses. Further scientific studies associated with transfersomes may lead to novel promising therapeutic approaches against many types of diseases.

## Figures and Tables

**Table 1:** Parameters and the testing methods of transfersomes.

Parameter	Method/Equipment
Vesicle size, size distribution Zeta potential	Dynamic light scattering (DLS) method by Malvern Zetasizer Electrophoretic mobility technique by Malvern Zetasizer
Vesicle morphology	DLS method, Photon correlation spectroscopy, Transmission electron microscopy
Number of vesicles for cubic mm	Hemocytometer and optical microscope
Entrapment efficiency	Directly or indirectly using high-performance liquid chromatography (HPLC) or spectrophotometric method $\%\mathrm{Entrapment}\;\mathrm{efficieny}\;=\;\frac{\mathrm{Amount}\;\mathrm{of}\;\mathrm{the}\;\mathrm{drug}\;\mathrm{entrapped}}{\mathrm{Total}\;\mathrm{amount}\;\mathrm{of}\;\mathrm{the}\;\mathrm{drug}\;\mathrm{added}}\times 100$ $\%\mathrm{Entrapment}\;\mathrm{efficiency}=\;\frac{\mathrm{Total}\;\mathrm{amount}\;\mathrm{of}\;\mathrm{the}\;\mathrm{drug}\;\mathrm{added}\;-\;\mathrm{Amount}\;\mathrm{of}\;\mathrm{the}\;\mathrm{free}\;\mathrm{drug}}{\mathrm{Total}\;\mathrm{amount}\;\mathrm{of}\;\mathrm{the}\;\mathrm{drug}\;\mathrm{added}}\times 100$
Drug content	Modified HPLC method using an ultraviolet detector, auto sample, column oven, pump and computerized analysis program depending upon the analytical method of the active pharmaceutical agent
Degree of deformability	Microporous filter with DLS, Transmission electron microscopy
Surface charge and charge density	DLS method by Malvern Zetasizer, Thin-layer chromatography
In-vitro drug release	Franz diffusion cell with cellulose membrane, Extrusion method
In-vitro skin permeation studies	Franz diffusion cell
In-vivo skin permeation ability	Confocal scanning laser microscopy (CSLM), Fluorescence microscopy, histological study used to determine the bioadhesive potential and retention of transfersomes in the skin [93]
Stability studies	DLS method, Transmission electron microscopy

**Table 2:** Examples of applications of transfersomes as a transdermal delivery system.

No.	Drug	Methods of Preparation and Formulation Details	Inference	Reference
1.	Insulin	Thin-film hydration technique (10 Pa), ethanolic SPC, SC (8.7 wt % SPC, 1.3 wt % SC, 8.5 vol % ethanol), triethanolamine–HCl buffer (pH 6.5) Subjected to intermediate-pressure homogenization or ultrasonication	Therapeutically significant hypoglycemia was induced in both mice and humans, with a good efficacy. as well as reproducibility	[54,74]
2.	Resveratrol	High-pressure homogenization technique (5 cycles, 1500 bar), PC, TW20, Plantacare^®^ 1200 UP, TW80	Antioxidant activity was not affected by coating, improved instability, bioavailability, solubility and safety	[79]
3.	Epigallocatechin-3-gallate (EGCG) and hyaluronic acid	Modified thin-film hydration method followed by high-pressure homogenization technique, chloroform:methanol (4:1 *v/v*), SPC, SC, hydration by PBS (pH 6.8); after removing the film, the mixture was passed through a high-pressure homogenizer (1000–1200 bars, 10 cycles)	Promising free radical-scavenging effect with considerably high skin permeation and deposition of EGCG	[100]
4.	Triamcinolone-acetonide	Thin-film hydration technique (10 Pa; 12 h), methanol/chloroform (1:1 *v/v*), SPC as an ethanolic solution (1:1 *w/v*), TW80 (9:11 *w/w*) relative to SPC, buffer (pH = 6.5), homogenized by sonication	Prolonged anti-inflammatory activity and 10-fold dose reduction to achieve the therapeutic level compared to the conventional formulation	[101]
5.	Corticosteroids Glucocortico steroids Hydrocortisone and dexamethasone	Conventional film method (10 Pa, 12 h), methanol/chloroform (1:1 *v/v*), SPC, TW20 (1:1 molar ratio relatively to SPC), buffer (pH = 6.5), homogenized by gentle sonication	Improved biological potency, prolonged effect and reduced therapeutic dosage	[102,105]
6.	Diclofenac sodium	Vortexing-sonication method and Rotary evaporation–sonication method (Despite the simple and less time-consuming nature of the vortexing method, rotary evaporation method was recommended), chloroform and methanol (2:1, *v/v*), EPC, SC, SDC, TW80, SP80, SP85, PBS (pH 7.4), sonicated in a bath sonicator	Maximum deformability was provided by vesicles consisted with tween 80 and more effective compared to bile salts and spans and significantly improved the in vitro skin delivery of the drug	[75]
		Thin-film hydration method, chloroform and ethanol, soya lecithin, SP20, SP60, SP80, PBS (pH 7.4)	Formulation with span 60 exhibited good entrapment efficiency and stability	[101]
		Rotary evaporation method, chloroform-methanol mixture (3:1), soya lecithin, CH, SP80	Significantly higher amount of cumulative permeation, steady state flux, permeability coefficient, residual drug into skin and promising stability over three months [56]	[102]
		Suspension homogenization (e.g., sonication), soy phosphatidylcholine, drug-to-lipid ratio between 1/4 and 1/9	Has shown the potential to replace combined oral/topical diclofenac administration in humans	[106]
7.	Celecoxib	modified handshaking method, chloroform:methanol (3:1 *v/v*), SPC, SDC, hydrated with PBS (pH 7.4), sonicated using bath sonicator	Proven to be therapeutically effective drug delivery system for treatment of rheumatoid arthritis	[92]
8.	Meloxicam [80]	Sonication method, chloroform: methanol (2:1 *v/v*), bilayer formation by EPC and either CH, SC, sodium oleate or dicetylphosphate in a molar ratio of 10:2	Significantly higher skin permeation	[40]
9.	Mefenamic acid	Modified handshaking technique and thin-film hydration technique, SPC, SP60, chloroform:ethanol (2:1), PBS (pH 7.4), stirred in orbitary shaker	Thin-film hydration method produced better results with highest drug content, spreadability and sustained drug release profile for 12 h	[107]
10.	Ketoprofen	Diractin^®^ (Trademark) Ketoprofen in transfersomes (nondisclosure of preparation methods and formulation details)	Superiorly effective in relieving the pain of knee osteoarthritis over a 6-week treatment period compared to placebo and comparatively fewer adverse events	[25,108]
			Proved to be efficacious in relieving pain from both eccentric muscle contractions and muscle over-exercise	[109]
11.	Ibuprofen	Lipid film hydration by rotary evaporation method, SPC, SP80, tween TW80, ethanol, PBS	Promising prolonged delivery system with reasonably good stability characteristics	[110]
		P90G, SP80 for F1, F2, F3 and F4 were 90:10, 85:15, 80:20 and 75:25 by direct mixing method	Result showed that F4 was the best transfersomes. With promising results for entrapment efficiency and increased in vitro skin permeation	[111]
12	Curcumin	Modified handshaking method, ethanol:chloroform (1:1), lecithin, TW80 and SP80, lecithin:surfactant ratio (95:05,85:15), solvents (ethanol, isopropyl alcohol), PBS (pH 7.4)	Exhibit potent anti-inflammatory properties with better permeation and bioavailability	[112,113]
13	Curcuma longa extract	Conventional rotary evaporation technique, chloroform:methanol (2:1), lecithin, TW80 and TW20, subjected to sonication	Promising photoprotective properties were observed with resulting improved skin properties (skin hydration and sebum content) with better skin penetration	[114,115]
14	Capsaicin	Conventional thin-film hydration method, P90G dissolved in ethanol, TW80, hydrated with distilled water, sonicated by bath sonicator	In vivo antiarthritic activity study exhibited superior inhibitory activity (in reducing arthritis and associated inflammations), enhanced permeability and better tolerance	[116]
		Thin-layer method, PC and the surfactant (SP80, TW80 and mixture of SP80 and TW80) (ratio 85:15) dissolved in dichloromethane, PBS (pH 7.4)	The transfersomal formulation using Tween 80 resulted in the best characteristics (the highest entrapment efficiency, the smallest particle size, the best deformability index and the highest penetration)	[72]
15	Vincristine	Dry-film and ultrasonic dispersing methods, Lecithin to SDC 70/20	Enhanced skin permeation and entrapment efficiency	[117]
		Dry-film and ultrasonic dispersing methods	Exhibited good lymph targeting ability	[118]
16	Tamoxifen	Thin-film hydration technique, chloroform, P90G, SP80, hydrated using triple distilled water; suspension was vortexed	Immense potential in management of psoriasis	[119]
17	Doxorubicin loaded hyaluronic acid modified transfersomes	Lipid film method, chloroform:ethanol (1:1, *v/v*), lecithin, GMS, SDC, PBS (pH 7.4), size reduction with sonication	Enhanced absorption by lymphatics and improved uptake by tumor cells	[120]
18	5-fluorouracil	Conventional rotary evaporation sonication method, chloroform and methanol (3:1 *v/v*), Lipoid S 100 (SPC), SP80, TW80 (95–80:5–20% *w/w* PC:surfactant), hydrated with 10% *v/v* ethanol in PBS (pH 6.5), subjected to sonication	Exhibited better skin penetration, as well as skin deposition, of the drug	[121]
19	Embelin	Thin-film hydration method, SP80 and TW80	Can be potentially used for the treatment of skin cancer	[122]
20	Felodipine	Vortexing-sonication method, SPC, EPC, TW80, SP80, lipid:edge activator (95:5)	The composition variation and the preparation method exhibited significant effects on the vesicles’ characteristics. Transfersomes rapidly, as well as noninvasively, permeated across the skin and achieved rapid therapeutic plasma drug levels at a lower dose	[77]
21	Telmisartan	Conventional rotary evaporation sonication method, chloroform:methanol (1:1), SPC was melted, dissolved in ethanol and SC, PBS (pH 6.4), sonicated using probe sonicator	Enhanced transdermal permeation and revealed to have prolonged release of the drug	[123]
22.	Eprosartan mesylate	Thin-film hydration technique, chloroform–methanol (2:1), P90G, SP80 and SDC, PBS (pH 7.4), sonication using a probe sonicator	Exhibited good entrapment of the drug and better transdermal flux	[90]
23.	Buspirone hydrochloride	Thin-layer evaporation technique, chloroform/methanol (2:1 *v/v*), EPC and TW80 (molar ratio 5:1); oleic acid was incorporated in some formulations as a transdermal permeation enhancer, hydrated with distilled water or hydroalcoholic solution, subjected to sonication	Promising physical stability, improved permeation and accurate dosing of potent hydrophilic drug	[85]
24.	Sertraline	Conventional rotary evaporation sonication method, ethanol, soya lecithin, SP80, hydrated with 7% *v/v* ethanol, subjected to sonication	Better antidepressant activity, with significantly higher advanced permeation characteristics	[124]
25	Tetanus-toxoid	Ethanolic solution of SPC was mixed with SDC (95:5; 90:10; 85:15; 80:20 and 75:25% *w/w*); PBS (pH 6.5) and the obtained suspension was pushed through a series of 0.45-, 0.22-, 0.10- and 0.05-µm polycarbonate membrane filters	Potential system of noninvasive topical immunization	[125,126]
26.	Lidocaine	Thin-film hydration method, SPC, CH, SC, SP80, and brij 35 different molar ratios were dissolved in a mixture of methanol:chloroform:ethanol)at (2:2:1) a *v/v/v* ratio, isotonic phosphate buffer (pH 5.8), subjected to sonication	Enhanced skin permeation with increased local anesthetic efficacy	[91,94]
27.	Repaglinide	Handshaking thin-film hydration method, methanol:chloroform (3:1), soy lecithin, SP80, SP60, SP40, TW80, PBS (pH 6.8), sonicated using bath sonicator	Improved topical delivery, site specificity and prolonged release of the antihyperglycemic agent	[127,128]
28.	Itraconazole	Thin-film hydration method, SPC, TW80, SP80, chloroform: methanol (3:1), phosphate buffer (pH 6.8), sonicated using for 30 min	Promising results with targeted and prolonged release of the drug	[129]
29.	Miconazole nitrate	Thin lipid film hydration technique, chloroform:methanol (2:1, *v/v*), soya lecithin, SP80, TW80, PBS (pH 7.4), sonicated using the sonicator digital sonifier	Showed a relatively higher antifungal activity than the standard commercial product	[83]
30.	Ketoconazole	Conventional solvent evaporation method, P90 G, ethanolic solution of Lipoid S100, TW80	Showed promising antimicrobial activity and excellent potential for the delivery of the drug	[93]
31	Raloxifene hydrochloride	Rotary evaporation method using chloroform:methanol (2:1), P90G:SP80 (85:15 *w/w*), hydrated with water, vesicle size reduction by sonication	Ex-vivo findings indicated a great potential for transdermal delivery	[130]
		Conventional thin-layer rotary evaporation method, chloroform and methanol (2:1, *v/v*), P90G, SDC, PBS (pH 6.5), subjected to sonication	Proved a significantly superior amount of drug permeation and deposition in the skin and identified to be a superior alternative compared to oral delivery [32]	[65]
32.	Tizanidine HCl	Conventional rotary evaporation method, chloroform, L-a-PC, CH, SP80, TW80, SDC, Brij 35, PBS, sonicated using a bath sonicator	Successively enhanced bioavailability and prolonged release of the drug; use for the treatment of spasticity	[56]
33.	Sildenafil Citrate	Thin-film hydration and sonication method, chloroform and methanol (1:1), P90 G, sodium lauryl sulphate, SP80, TW80, cetrimide, PBS (pH 5.8), sonicated using probe sonicator	Treatment for erectile dysfunction. Improved transdermal permeation and bioavailability with reduced dose administration frequency	[102,131,132]
34.	Interleukin-2 and Interferon-α	PC, SC, PBS	Agents were incorporated in the transfersomes for immunotherapy in sufficient concentrations and in their biological active form	[133]
35.	Cyclodextrin-colchicine complex	Conventional rotary evaporation sonication method, chloroform:methanol (2:1), SPC, CH, SP80, SP60, hydrated with drug, drug-cyclodextrin complex or fluorescence marker solution in ethanol, subjected to sonication	Showed 6-fold increase in transdermal flux and better and sustained antigout activity in rats. Enhanced skin accumulation, prolonged drug release, and improved the site-specificity of colchicine	[134]
	Colchicine		Increased skin penetration	[135]
36.	Caffeine	Thin-film hydration method, chloroform, DLPC:DPGluc:CH (35:7:7), hydrated with caffeine solution (2% in water)	Showed significantly greater permeation across the stratum corneum and significant enhancement of hydrophilic drug caffeine penetration into hair follicles	[136]
37.	Minoxidil and caffeine	Modified thin-film hydration method, SPC, TW20, TW80, lecithin was mixed with various amounts of edge activators and dissolved in chloroform	Increasing the ratio of polysorbate enhanced the entrapment efficiency of minoxidil and caffeine. Topical application of this formulation more effectively promoted the hair growth in rats compared to the commercial product	[137]
38.	Tacrolimus	Thin lipid film hydration method, SPC, SDC, chloroform/methanol (2:1 *v/v*), hydration medium phosphate buffer, pH 7.2	Better antipsoriatic activities compared to liposomes due to better skin permeations	[138]
39.	Estradiol	Bath sonication and homogenization by manual extrusion, DPPC, SP80, TW80, SDC	Superior estradiol skin delivery compared to traditional liposomes	[139]

PC: phosphatidylcholine:lecithin, SPC: soy phosphatidylcholine, EPC: egg phosphatidylcholine, DPPC: dipalmitoylphosphatidylcholine, DLPC: dilauroyl L-α-phosphatidylcholine, P: phospholipon, CH: cholesterol, PBS: phosphate-buffered saline, SP: span, TW: Tween, SC: sodium cholate, SDC: sodium deoxycholate, DPGluc: decyl polyglucoside and GMS: glycerol α-monostearate.
